# A novel method for transmitting southern rice black-streaked dwarf virus to rice without insect vector

**DOI:** 10.1186/s12985-017-0815-4

**Published:** 2017-08-15

**Authors:** Lu Yu, Jing Shi, Lianlian Cao, Guoping Zhang, Wenli Wang, Deyu Hu, Baoan Song

**Affiliations:** State Key Laboratory Breeding Base of Green Pesticide and Agricultural Bioengineering, Key Laboratory of Green Pesticide and Agricultural Bioengineering, Ministry of Education, Guizhou University, Guiyang, 550025 China

**Keywords:** SRBSDV, Bud-cutting method, Transmission efficiency, PCR, Proteomics

## Abstract

**Background:**

Southern rice black-streaked dwarf virus (SRBSDV) has spread from the south of China to the north of Vietnam in the past few years, and has severely influenced rice production. However, previous study of traditional SRBSDV transmission method by the natural virus vector, the white-backed planthopper (WBPH, *Sogatella furcifera*), in the laboratory, researchers are frequently confronted with lack of enough viral samples due to the limited life span of infected vectors and rice plants and low virus acquisition and inoculation efficiency by the vector. Meanwhile, traditional mechanical inoculation of virus only apply to dicotyledon because of the higher content of lignin in the leaves of the monocot. Therefore, establishing an efficient and persistent-transmitting model, with a shorter virus transmission time and a higher virus transmission efficiency, for screening novel anti-SRBSDV drugs is an urgent need.

**Methods:**

In this study, we firstly reported a novel method for transmitting SRBSDV in rice using the bud-cutting method. The transmission efficiency of SRBSDV in rice was investigated via the polymerase chain reaction (PCR) method and the replication of SRBSDV in rice was also investigated via the proteomics analysis.

**Results:**

Rice infected with SRBSDV using the bud-cutting method exhibited similar symptoms to those infected by the WBPH, and the transmission efficiency (>80.00%), which was determined using the PCR method, and the virus transmission time (30 min) were superior to those achieved that transmitted by the WBPH. Proteomics analysis confirmed that SRBSDV P1, P2, P3, P4, P5–1, P5–2, P6, P8, P9–1, P9–2, and P10 proteins were present in infected rice seedlings infected via the bud-cutting method.

**Conclusion:**

The results showed that SRBSDV could be successfully transmitted via the bud-cutting method and plants infected SRBSDV exhibited the symptoms were similar to those transmitted by the WBPH. Therefore, the use of the bud-cutting method to generate a cheap, efficient, reliable supply of SRBSDV-infected rice seedlings should aid the development of disease control strategies. Meanwhile, this method also could provide a new idea for the other virus transmission in monocot.

**Electronic supplementary material:**

The online version of this article (doi:10.1186/s12985-017-0815-4) contains supplementary material, which is available to authorized users.

## Background

Belonging to the genus *Fijivirus* (family *Reoviridae*), southern rice black-streaked dwarf virus (SRBSDV) is the causal agent of a rice dwarfing disease that was first discovered and named in China. SRBSDV was recognized as a new species in 2008 [1]. SRBSDV has spread from the south of China to the north of Vietnam in the last few years, and has severely affected rice production [2–4]. In 2009, SRBSDV infected nearly 741,000 acres of rice and rice farms covering 100,000 acres in total reported crop failure, which was a significant loss in China [5]. Meanwhile, in 2010, about 2.97 million acres in China were effected by SRBSDV [6]. To try to combat this disease, researchers are actively screening for effective anti-SRBSDV drugs and investigating preventive measures for controlling SRBSDV. However, the lack of sufficient viral samples due to the limited life span of infected vectors and rice plants and the vector’s low virus acquisition and inoculation rate is hampering this research effort. To generate viral samples, researchers have to successively collect rice plants and white-backed planthopper (WBPH, *Sogatella furcifera*) from paddy fields infected with SRBSDV, which is time-consuming, expensive and, most importantly, this method does not ensure a timely supply of SRBSDV-infected vectors or infected rice plants [7, 8]. Therefore, there is an urgent need to establish an efficient and persistent-transmitting model, with a shorter virus transmission time and a higher virus transmission efficiency, to screen novel anti-SRBSDV drugs. Furthermore, SRBSDV not only infects rice but also a number of other graminaceous plants, such as *Echinochloa crusgalli* L., *Zea mays* L., *Paspalum distichum* L., and *Alopecurus aequalis* Sobol., and cyperaceous plants, such as *Juncellus serotinus* (Rottb.) C. B. Clarke and *Cyperus difformis* L. [9].

In the laboratory, SRBSDV is traditionally transmitted by the WBPH, a long-distance migratory pest, via the method of simulating the natural environment [2, 10]. When infected with SRBSDV, the rice symptoms often develops stunted stems, dark green and twisted leaves, white waxy swellings on the leaf veins and so on [11]. However, the symptoms are not obvious at the beginning of the infection, so several methods, such as polymerase chain reaction (PCR), enzyme-linked immunosorbent assay (ELISA), and reverse transcription loop-mediated isothermal amplification assay, have been applied to diagnose SRBSDV infection. Meanwhile, PCR denaturing gradient gel electrophoresis (PCR-DGGE) has been used to measure at different temperatures, how SRBSDV’s transmission efficiency and their characteristics among various host plants change [8, 12–14]. In addition, our group has previously reported a novel method relied on rice suspension cells so that we can test antiviral compounds against SRBSDV; the transmission efficiency of SRBSDV was determined using the real time quantitative PCR (RT-qPCR) [15].

In this study, we firstly reported a novel method for transmitting SRBSDV in rice using the bud-cutting method to generate a reliable supply of rice infected with SRBSDV for screening novel anti-SRBSDV drugs. The results showed that SRBSDV could be successfully transmitted via the bud-cutting method and plants infected SRBSDV exhibited the symptoms were similar to those transmitted by the WBPH. Meanwhile, the transmission efficiency of SRBSDV in rice was investigated via the PCR method and the results demonstrated that the bud-cutting method was an efficient persistent-transmitting method with a shorter virus transmission time (30 min) and a higher virus transmission efficiency (> 80.00%). Moreover, proteomics analysis demonstrated that SRBSDV could also replicate in rice in the whole growth period. To our knowledge, it is the first report on the SRBSDV transmission in rice using the bud-cutting method.

## Methods

### Material preparation

Rice plants, after elongation stage that had obvious tumor-like protrusion symptoms and checked the SRBSDV via the RT-qPCR method, studied herein were acquired in Libo city, Guizhou province, southern China in the autumn of 2015. The rice samples were frozen in liquid nitrogen and ground to obtain a fine powder. Then, the powder (approximately 30 g) was soaked in 100 mL 0.2 M PBS buffer (pH 5.8; consists of 0.2 M PBS, 10 mM 2-(*N*-morpholino)ethanesulfonic acid, and 100 μM acetosyringone) for 30 min and filtered with gauze to obtain the crude SRBSDV extraction buffer.

### SRBSDV transmission

**Fig. 1:**
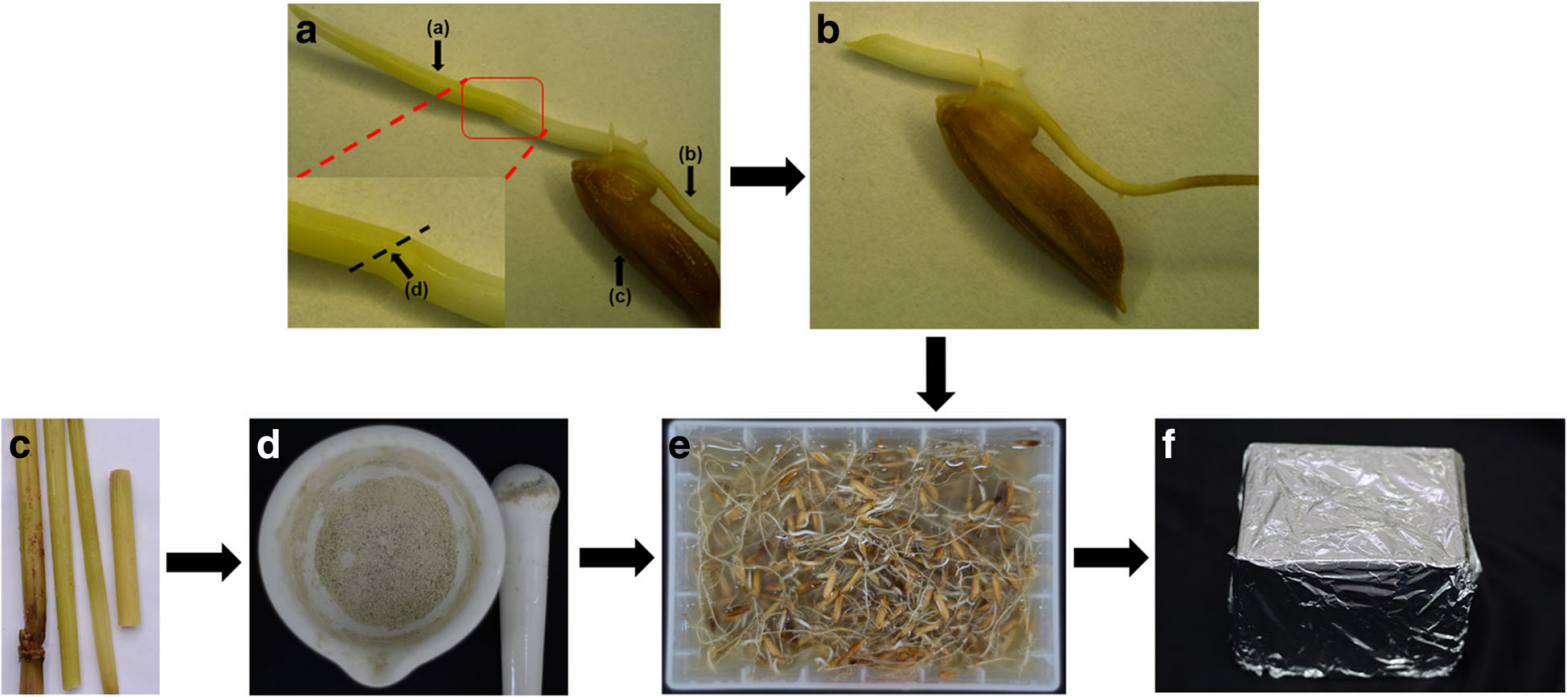
The SRBSDV transmission process using the bud-cutting method. **a** The germinated rice seed. (**a**) The germ, (**b**) The radicle, (**c**) The cotyledon, (**d**) The position (a cyclic structure) for cutting bud at a 45° angle approximately 0.5 cm from the base of the bud. **b** The bud-cutting rice seed. **c** The rice stems which were had obvious tumor-like protrusion symptoms. **d** The rice stems were frozen in liquid nitrogen and ground to obtain a fine powder. **e** The treated seedlings were soaked in the crude SRBSDV extract for 30 min to inoculate the seedlings with SRBSDV. **f** The seedlings were incubated in the dark at 28–30 °C for 3 days

Rice seeds (*Oryza sativa* L., Fengyouxiangzhan) were soaked in water for approximately 2 days at room temperature. After breaking the seed coat, the seeds were pre-germinated at 40 °C for approximately 3 days in an illuminated incubator until a bud of approximately 1.0 cm in length had developed. The bud was cut at a 45° angle approximately 0.5 cm from the base of the bud (Fig. 1). Then, the treated seedlings were soaked in the crude SRBSDV extract for 30 min to inoculate the seedlings with SRBSDV before incubating the seedlings in the dark at 28–30 °C for 3 days*.* The rice seedlings were then planted in a soil matrix and incubated for 25 days at a relative humidity of 50%, a temperature of 28–30 °C, and light/dark cycles of 14/10 h. Then, samples were collected from the rice plants and maintained at −80 °С until needed for determining the SRBSDV transmission efficiency and characterizing the SRBSDV proteins. Finally, the rice seedlings were planted out in the experimental fields to observe any symptoms of disease.

### RNA extraction, cDNA synthesis, and PCR analysis

According to the instructions of the manufacturer’s instructions, total RNAs were extract from the samples via the use of a Trizol reagent kit (TaKaRa, Dalian, China). The purity and concentration of RNA was determined with an ultraviolet spectrophotometer (ACTGene, Piscataway, NJ, USA) and total RNA purity was estimated by calculating OD_260_/OD_280_. And the concentration of total RNA was calculated according to the dilution ratio and the value of OD_260_.

cDNAs were also synthesized using TaKaRa’s cDNA synthesis kit. Random primers (0.5 μL) and total RNA (3000 ng/μL, 1 μL) were added to H_2_O (4.75 μL) and heated for an hour at 42 °C and then 15 min at 70 °C. Then, the solution was rapidly placed on ice to cool for 2 min. After that, 2 μL of 5 × Moloney murine leukemia virus (M-MLV) buffer, 500 nL of 10 mM dNTP, 250 nL of RNase Inhibitor (RRI), and 500 nL of M-MLV Reverse Transcriptase were added to the above solution. Finally, in the thermocycler, the PCR amplification conditions were set as: 10 min at 30 °C, an hour at 42 °C, and 15 min at 70 °C.

A final reaction volume of 20 μL, consists of 2 μL of 10 × PCR buffer, 1.6 μL of dNTP (2.5 mM), 1.6 μL of MgCl_2_ (2.5 mM), 200 nL of rTaq, 800 nL of each S7–1 primer (10 mmol; forward primer, 5′-GGAATTCATGGATAGACCTGCTCGA-3′; reverse primer, 5′-CGGGATCCTTAAGATGATGGAGATTC-3′) or S9–1 primer (10 mmol; forward primer, 5′-GGAATTCATGGCAGACCAAGAGCGT-3′; reverse primer, 5′-CGGGATCCTCAAACGTCCAATTTAAG-3′), which were designed using the Primer Premier 5.0 software (PREMIER Biosoft International, USA) according the reported complete genome sequence of SRBSDV [16], 2 μL of cDNA, and 11 μL of H_2_O, was produced for PCR amplifications. The amplification conditions were set at 94 °C for 120 s and followed by 35 cycles of 30 s at 94 °C, 30 s at 58 °C, and 70 s at 72 °C, the next stage was extension at 72 °C and 12 °C for 10 min, respectively, to realize a dissociation curve. The amplicons were sequenced at Sangon Corporation (Shanghai, China) and the results were compared with the known sequences deposited in the National Center for Biotechnology Information (NCBI, https://www.ncbi.nlm.nih.gov/) database using the Standard Nucleotide BLAST program.

After PCR analysis, the whole PCR reaction product was electrophoresed for 15 min on a 1.5% agarose gel. The transmission efficiency was calculated using the PCR results. The experiment was replicated three times.

### Protein extraction, mass spectrometry, and protein identification

The protein extraction procedure was based on the phenol extraction method [17, 18]. First, each pooled sample (approximately 1.5 g) was frozen in situation filled with liquid nitrogen. Then they were ground into fine powder. 5 mL of protein extraction buffer of ice-cold temperature, which consists of 0.5 M Tris-HCl (pH 7.5), 0.7 M sucrose, 0.1 M KCl, 50 mM EDTA, and 40 mM dithiothreitol (DTT), was provided for suspending at room temperature for 15 min. Add 5 mL of Tris-phenol to the suspension before shaking for 30 min and then centrifuging the suspension at 8000 *g* at 4 °C for 5 min. Collect the upper phenolic phase and add equal extraction buffer to the supernatant. Then, add 4 volumes of 0.1 M ammonium acetate dissolved in methanol to the supernatant before leaving it overnight at −20 °C for the aim of protein precipitation. Next, discard the supernatant after centrifugation 8000 *g* at 4 °C for 10 min, and then washed the pellet three times with acetone at 4 °C. Finally, the pellet was dried in a vacuum drier for 2 h before solubilizing in 100 μL of the rehydration solution (8 M (*w*/*v*) urea, 0.1 M (*w*/*v*) Tris, 10 mM DTT). Moreover, the protein concentration was measured using the Bradford method [19].

Through the use of a LC-MS/MS system, the total peptides from the proteins extracted from rice were measured [20]. A mass spectrometer (5600 Triple TOF MS) coupled to a Nano-Liquid Chromatogram (Eksigent, Dublin, CA) was used to perform all the analytics. First, 8 μL of peptides (1 μg/μL) were injected automatically into a six-port valve and desalted for 10 min by applying a ChromXP Trap column (Nano LC TRAP Column) with the speed of 2 μL/min. Second, the retained peptides were separated via a gradient elution mode using an analytical column-Nano LC C_18_ reversed-phase column (3C_18_-CL, 75 μm × 15 cm) at the speed of 300 nL/min, with 95% solvent B and 95% solvent A. The former consists of 95% acetonitrile (ACN), 5% HPLC grade water and 0.1% *v*/v formic acid (FA), while the latter consists of 5% ACN, 95% HPLC grade water and 0.1% *v*/v FA. Third, Analyst® Software (TF 1.6) was used to operate the 5600 Triple TOF MS and enable to switch between TOF-MS and Product Ion acquisition automatically. Ultimately, the data were collected after selecting a range of 300–1600 m/z. In addition, a *β*-galactosidase digest standard was used to recalibrate the mass spectrometer when the flow path run of each sample was completed and a high voltage of 2.3 kV was applied for stable spray operation. The use of Analyst® Software (TF 1.6; Foster City, CA, USA) could control the LC pump, the automatic mass spectra acquisitions and mass spectrometer.

In this study, raw data from shotgun data acquisition (Wiff. files) were analyzed and identified through the use of Protein Pilot 4.5 software. The UniProt (release 2016–12) database, restricted to SRBSDV, was used to search the MS/MS spectra using the Andromeda search engine [21]. The sequences of all proteins were retrieved using their accession numbers in FASTA format from Uniprot database (http://www.uniprot.org/).

## Results

### Symptoms

**Fig. 2:**
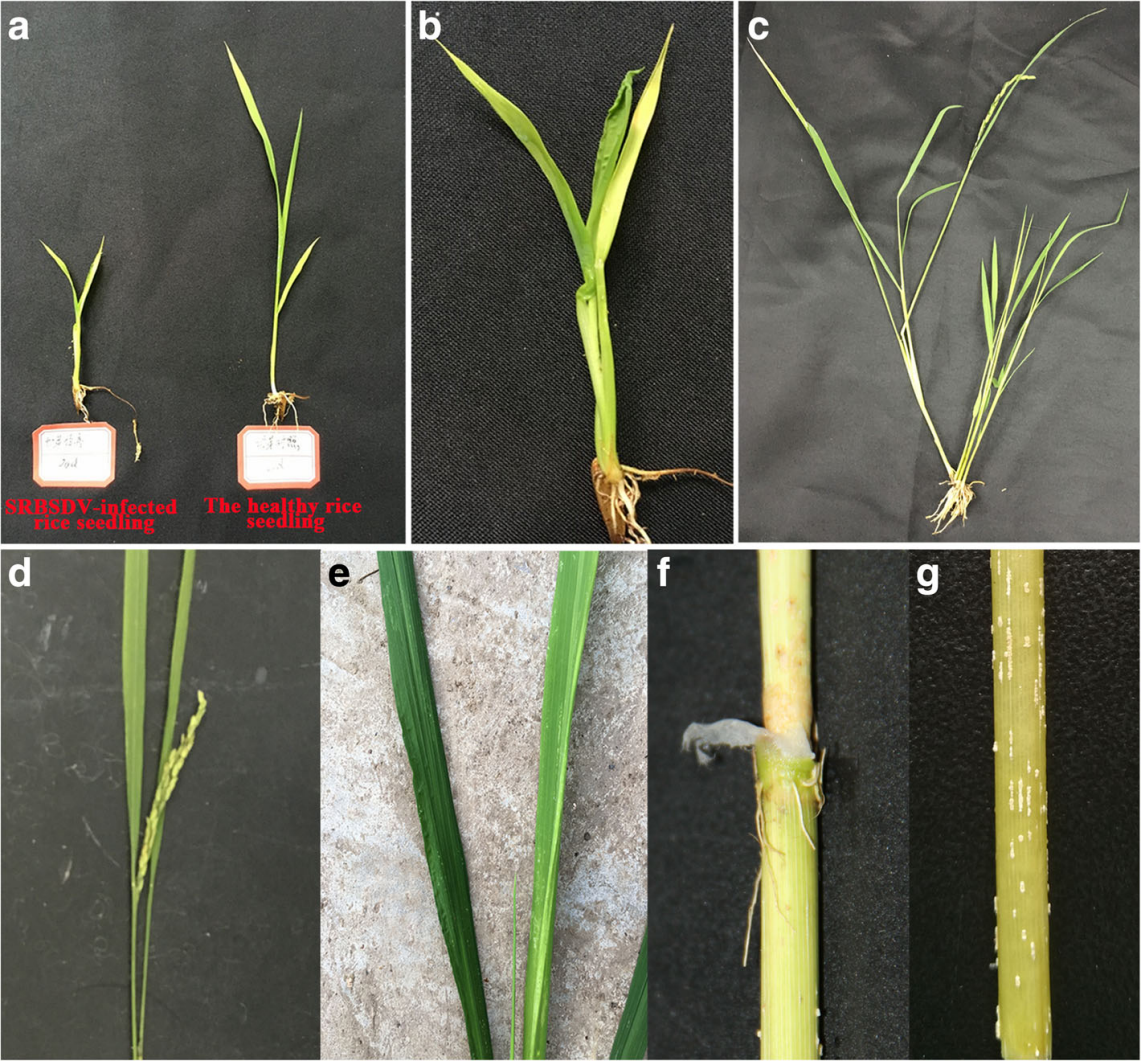
Symptoms of SRBSDV disease in rice infected with SRBSDV using the bud-cutting method. **a** Infected seedling showing symptoms of dwarfism (left); uninfected seedling (right). **b** Infected seedling with stiff leaves. **c** Plant infected when tillering showing excessive dwarfing and tillering. **d** Plant infected at the elongation stage: although growth has not been stunted, the spikes are small with barren grains and low grain weight. **e** Leaves of a diseased plant: short, dark green, and rigid, with ruffles near the leaf base. **f** Aerial rootlets at the stem node. **g** Stem with small, streaked, white tumor-like protrusions (1–2 mm)

Plants infected at the early seedling stage exhibit dwarfism (less than half the normal height) and stiff leaves (Fig. 2a and b). Infection at the early tillering stage exhibited excessive dwarfing and tillering (Fig. 2c). When infected at the elongation stage, plants developed small spikes with barren grains and grain weight was low (Fig. 2d). The infected leaves were quite short, dark green, and rigid. Moreover, ruffles could be seen on the surfaces of the upper leaves (Fig. 2e). After elongation, aerial rootlets appeared at nodes (Fig. 2f); small, streaked, black or white tumor-like protrusions (1–2 mm in size) appeared on the stems (Fig. 2g).

### Determination of SRBSDV transmission efficiency

A comparison of the infected sample sequences and the known SRBSDV S7–1 sequence (accession number: KM576880) and SRBSDV S9–1 sequence (accession number: KJ476862.1) revealed that sequence identity was 99%. The transmission efficiency of SRBSDV to rice plants via the bud-cutting method was calculated using the PCR results and the PCR electrophoresis diagram can be found in Additional file 1: Figure S1. As shown in Table 1, the SRBSDV transmission efficiency in infected rice seedlings via the bud-cutting method was 90.00%, 91.67%, and 88.89% in trials 1, 2, and 3, respectively.

**Table 1:** SRBSDV transmission efficiency of the bud cutting method in rice seedlings

Trial	No. of rice seedlings tested	No. of rice seedlings infected	Transmission efficiency (%)
1	10	9	90.00
2	12	11	91.67
3	9	8	88.89

### Protein identification

The SRBSDV P1, P2, P3, P4, P5–1, P5–2, P6, P8, P9–1, P9–2, and P10 proteins, with the confidence level values of 66%, 66%, 95%, 99%, 99%, 99%, 99%, 99%, 99%, 99%, and 99%, respectively (Table 2), were identified in the infected rice seedlings using a proteomics approach, which demonstrated that SRBSDV was successfully transmitted to rice plants via the bud-cutting method. The detailed data of the peptides hits to each protein was shown in Additional file 2.

**Table 2:** Identification of SRBSDV proteins in the viruliferous rice seedlings

NO.	Protein ID	Protein name	Species	Unused	Conf.
1	E4WKV7_9REOV	P1 protein	9REOV	0.53	66
2	J9UCP0_9REOV	P2 protein	9REOV	1.12	66
3	M4GP49_9REOV	P3 protein	9REOV	1.87	95
4	E4WKW7_9REOV	P4 protein	9REOV	4.18	99
5	J9UM56_9REOV	P5–1 protein	9REOV	2.51	99
6	H9BJU8_9REOV	P5–2 protein	9REOV	2.13	99
7	N0A5Y4_9REOV	P6 protein	9REOV	6.12	99
8	A0A024CLV9_9REOV	P8 protein	9REOV	2.36	99
9	A0A0C4W4C8_9REOV	P9–1 protein	9REOV	2.18	99
10	N0A4C0_9REOV	P9–2 protein	9REOV	2.04	99
11	G0ZCH9_9REOV	P10 protein	9REOV	2.17	99

## Discussion

Researchers studying SRBSDV–insect vector–host plant interactions are frequently hampered by a lack of sufficient viral samples owing to the limited life span of the infected vectors and rice plants and the low virus acquisition and inoculation rate of the WBPH vector. Previous studies have reported that the minimum virus acquisition period ranges from 2 to 8 min [7, 14], that an inoculation access period of 30 min is required for not only WBPH nymphs but also WBPH adults, and that the period of virus’s circulative transmission ranges from 3 to 14 days. Furthermore, the major of infected individuals transmit virus during intermittent periods, which range between 2 and 6 days [7, 14]. Meanwhile, Li and coworkers reported that the transmission efficiencies of SRBSDV by macropterous and brachypterous WBPH adults from infected rice plants to healthy rice seedlings were 23.7% at 15 °C, 53.6% at 25 °C, and 7.3% at 35 °C [8]. By contrast, the virus transmission abilities of SRBSDV in rice plants using the bud-cutting method were determined in detail in this study and the results were confirmed that the bud-cutting method was an efficient persistent-transmitting method with a shorter virus transmission time (30 min) and a higher virus transmission rate (> 80.00%).

The rice disease caused by SRBSDV produces symptoms similar to those induced by rice black-streaked dwarf virus (RBSDV) at different growth stages of the host. In this study, we obtained similar disease symptoms to those observed on rice plants infected with SRBSDV by the WBPH vector (Fig. 1). These symptoms can show as dwarfism and stiff leaves at the early seedling stage; small spikes, barren grains, abnormally short and dark green leaves with ruffles at the elongation stage; aerial rootlets and branching at nodes, and small streaked waxy galls on stems, which agrees with the findings reported by previous studies [1, 11].

Some studies have indicated that SRBSDV is composed of icosahedral particles, consisting of 10 segments, which are defined as S1 to S10 based on size mainly ranging from 4.5 to 1.4 kb [2, 12, 22]. When compared with RBSDV, SRBSDV encodes six putative structural proteins (P1, P2, P3, P4, P8, and P10) at least [23, 24], which respectively refer to a putative RNA-dependent RNA polymerase, a core protein, a putative capping enzyme, an outer-shell B-spike protein, a putative core protein, and a major outer capsid protein [25–29]. Moreover, SRBSDV encodes five putative nonstructural proteins (P6, P7–1, P7–2, P9–1, and P9–2), among which P6 refers to a viral RNA-silencing suppressor and tubules in nonhost insect cells [30]. Through forming intracellular viroplasms, P9–1 plays a necessary role in virus’s early life [31]. Herein, we adopted a proteomics approach to identify the SRBSDV P1, P2, P3, P4, P5–1, P5–2, P6, P8, P9–1, P9–2, and P10 proteins in the infected rice seedlings, which further validated the successful transmission of SRBSDV using the bud-cutting method. Unfortunately, in our present study, SRBSDV P7–1 and P7–2 proteins failed to detect in the infected rice seedlings. The undetectable expression level of P7–1 and P7–2 proteins might result from lacking of the transmitting vector. SRBSDV P7–1 protein, a virus movement protein, was recently reported to induce the formation of tubular structures in insect cells and played a key role in virus transmission via the insect vector [32]. Interestingly, our previous work showed that SRBSDV P7–1 protein could be detected in rice plants infected SRBSDV via the insect vector [32]. By contrast, in this study, SRBSDV was successfully transmitted to rice plants without insect vector and, therefore, SRBSDV P7–1 protein could not be detected in the infected rice seedlings via the proteomics method. Meanwhile, RBSDV P7–2 protein and its counterparts were considered to be involved in virus multiplication in plants or viral pathogenicity on plants [33]. However, literature reported that SRBSDV P7–2 protein was failed to detect in either SRBSDV infected plants or insects over the past two decades [34]. The result was consistent with our present study result, in which SRBSDV P7–2 protein was not yet identified in SRBSDV-infected hosts.

## Conclusions

Our findings showed that SRBSDV could be successfully transmitted from infected to healthy rice seedlings using the bud-cutting method and this method resulted in a shorter virus transmission time and a higher transmission efficiency than transmitted by the WBPH. The use of the bud-cutting method to generate a cheap, efficient, reliable supply of SRBSDV-infected rice seedlings should aid the development of disease control strategies. And this method also could provide a new idea for the other virus transmission in monocot.

## Additional files

Additional file 1: Fig. S1. SRBSDV detection by the PCR method. (A) M: DL2000 maker, Lanes 1–10: The S9–1 gene in rice plants infected with SRBSDV using the bud-cutting method; (B) M: DL2000 maker, Lanes 1–12: The S9–1 gene in rice plants infected with SRBSDV using the bud-cutting method; (C) M: DL2000 maker, Lanes 1–9: The S7–1 gene in rice plants infected with SRBSDV using the bud-cutting method. (DOCX 275 kb)

Additional file 2: The detailed data of the peptides hits to each protein. (XLSX 1578 kb)

## Abbreviations

ACN: Acetonitrile
DTT: Dithiothreitol
ELISA: Enzyme-linked immunosorbent assay
FA: Formic acid
M-MLV: Moloney murine leukemia virus
NCBI: National center for biotechnology information
PCR: Polymerase chain reaction
PCR-DGGE: PCR denaturing gradient gel electrophoresis
RRI: RNase inhibitor
RT-qPCR: Real time quantitative PCR
SRBSDV: Southern rice black-streaked dwarf virus
WBPH: White-backed planthopper
