# Supplementary material for: A novel method for transmitting southern rice black-streaked dwarf virus to rice without insect vector
Source: Virol J. 2017 Aug 15;14:155. doi: 10.1186/s12985-017-0815-4 (PMC5558718; doi:10.1186/s12985-017-0815-4)
Supplement: Supplementary file 1 — SRBSDV detection by the PCR method. (A) M: DL2000 maker, Lanes 1–10: The S9–1 gene in rice plants infected with SRBSDV using the bud-cutting method; (B) M: DL2000 maker, Lanes 1–12: The S9–1 gene in rice plants infected with SRBSDV using the bud-cutting method; (C) M: DL2000 maker, Lanes 1–9: The S7–1 gene in rice plants infected with SRBSDV using the bud-cutting method. (DOCX 275 kb) [file 12985_2017_815_MOESM1_ESM.docx]

Supplementary material

A novel method for transmitting southern rice black-streaked dwarf virus to rice without insect vector

Lu Yu, Jing Shi, Lianlian Cao, Guoping Zhang, Wenli Wang, Deyu Hu, Baoan Song *

State Key Laboratory Breeding Base of Green Pesticide and Agricultural Bioengineering; Key Laboratory of Green Pesticide and Agricultural Bioengineering, Ministry of Education, Guizhou University, Guiyang 550025, China; E-mails: yuji570@163.com (L.Y.); 18985418398@163.com (J.S.); 15585293343@163.com (L.C.); hbzgp-1@163.com (G.Z.); wangwenli0208@163.com (W.W.); dyhu@gzu.edu.cn (D.H.)

*****Correspondence: songbaoan22@yahoo.com; Tel.: +86-0851-83620521, Fax: +86-0851-83622211.


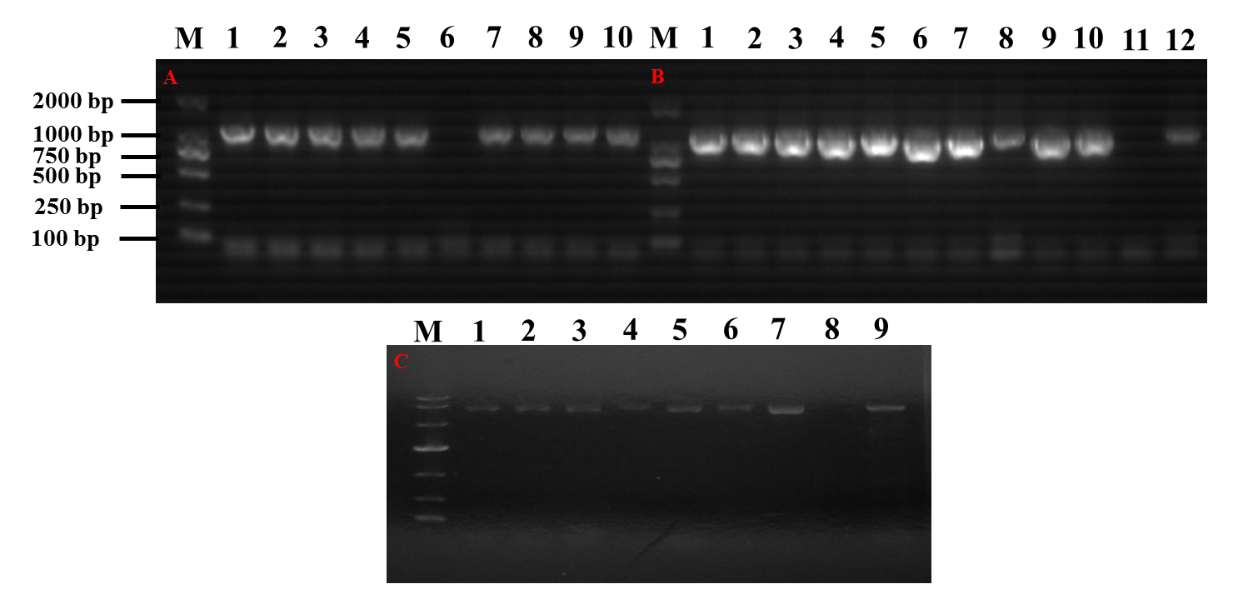


**Figure 1.** SRBSDV detection by the PCR method. (**A**) M: DL2000 maker, Lanes 1–10: The S9-1 gene in rice plants infected with SRBSDV using the bud-cutting method; (**B**) M: DL2000 maker, Lanes 1–12: The S9-1 gene in rice plants infected with SRBSDV using the bud-cutting method; (**C**) M: DL2000 maker, Lanes 1–9: The S7-1 gene in rice plants infected with SRBSDV using the bud-cutting method.
